# Contrasting patterns of population structure of Bulwer’s petrel (*Bulweria bulwerii*) between oceans revealed by statistical phylogeography

**DOI:** 10.1038/s41598-023-28452-z

**Published:** 2023-02-02

**Authors:** Mónica C. Silva, Paulo Catry, Joël Bried, Kazuto Kawakami, Elizabeth Flint, José P. Granadeiro

**Affiliations:** 1 Departamento de Biologia Animal, Faculdade de Ciências, cE3c - Centre for Ecology, Evolution and Environmental Changes and CHANGE – Global Change and Sustainability Institute, Universidade de Lisboa, 1749-016 Campo Grande, Lisbon, Portugal; 2 MARE - Marine and Environment Sciences Centre / ARNET - Aquatic Research Network, Ispa - Instituto Universitário, Rua Jardim do Tabaco 34, 1149-041 Lisboa, Portugal; 3 Departamento de Oceanografia E Pescas, IMAR and LARSyS Associated Lab, Centro Okeanos, MARE (Marine and Environmental Sciences Centre), Universidade Dos Açores, 9901-862 Horta, Azores, Portugal; 4 Laboratory of Wildlife Ecology, Forestry and Forest Products Research Institute (FFPRI), 1 Matsunosato, Tsukuba, Ibaraki 305-8687 Japan; 5 Pacific Islands Refuges and Monuments Office, US Fish and Wildlife Services, Washington, DC USA; 6 Departamento de Biologia Animal, Faculdade de Ciências, CESAM - Centre for Environmental and Marine Studies, Universidade de Lisboa, 1749-016 Campo Grande, Lisbon, Portugal; 7 Present Address: 64200 Biarritz, France

**Keywords:** Evolution, Zoology

## Abstract

The patterns of population divergence of mid-latitude marine birds are impacted by only a few biogeographic barriers to dispersal and the effect of intrinsic factors, such as fidelity to natal colonies or wintering grounds, may become more conspicuous. Here we describe, for the first time, the phylogeographic patterns and historical demography of Bulwer’s petrel *Bulweria bulwerii* and provide new insights regarding the drivers of species diversification in the marine environment. We sampled Bulwer’s petrels from the main breeding colonies and used a statistical phylogeography approach based on surveying nuclear and mitochondrial loci (~ 9100 bp) to study its mechanisms of global diversification. We uncovered three highly differentiated groups including the Western Pacific, the Central Pacific and the Atlantic. The older divergence occurred within the Pacific Ocean, *ca*. 850,000 ya, and since then the W Pacific group has been evolving in isolation. Conversely, divergence between the Central Pacific and Atlantic populations occurred within the last 200,000 years. While the Isthmus of Panama is important in restricting gene flow between oceans in Bulwer’s petrels, the deepest phylogeographic break is within the Pacific Ocean, where oceanographic barriers are key in driving and maintaining the remarkable structure found in this highly mobile seabird. This is in contrast with the Atlantic, where no structure was detected. Further data will provide insights regarding the extent of lineage divergence of Bulwer’s petrels in the Western Pacific.

## Introduction

Reconstructing the diversification history of species and identifying the main drivers of population differentiation in widely distributed species is key to understand population connectivity and how biodiversity is generated and maintained, particularly in the face of a rapidly changing world. Empirical statistical phylogeographic studies have been unveiling the primary drivers of global diversification of marine species, particularly those of high latitudes^1–7^. Mechanisms of population divergence of seabirds with pan-tropical distributions however are not as well understood^8–11^. Historical vicariant events, such as the emergence of the Isthmus of Panama in the late Pliocene, can create phylogeographic structure in species that previously occupied a single region, effectively isolating lineages and promoting speciation^12–15^. Other oceanic features, such as the Eastern Pacific Barrier, are also emerging as important barriers to gene flow in a variety of taxa^9,10,16^, despite the ability of marine species to disperse long distances. Changes in the oceanic environment driven by historical climatic changes have also been proposed as important drivers of divergence in marine taxa^1,2,5,14,17,18^, although their effect in mid-latitude species is not as clear.

Cryptic, intrinsic barriers, such as strong philopatry, which is characteristic of most pelagic seabirds, phenological differences driven by variation in non-breeding distributions and adaptation to local environmental conditions, may also play an important role on population divergence, sometimes even in the presence of gene flow^6,8,19–21^.

Bulwer’s petrel *Bulweria bulwerii* is a useful model to study the impact of physical and non-physical barriers in the diversification history of marine species. This small (*ca*. 100 g), all-dark procellariiform species is distributed in the tropical and sub-tropical oceanic islands of the Pacific and Atlantic oceans. It has also been recently discovered breeding on Round I. off Mauritius in the Indian Ocean, but the population is very small^22^. The species occurs in deep sea year-round^23–25^. It breeds on small oceanic islands for close to five months of the year, a period during which it behaves as a central place forager due to the constraints of incubation of the single egg and chick provisioning^25^. Individuals perform long migrations between their breeding and non-breeding areas. In the Atlantic, wintering areas include the southern equatorial regions^23,24^. In the Pacific, wintering areas are not as well known, but W Pacific populations are thought to migrate SW into the Indian Ocean, while Central Pacific populations are thought to move SE^22,26^. Like most procellariiform seabirds, the species is philopatric^27^. Although the Bulwer’s petrel population in the NW Hawaiian Islands was thought to be the largest with almost 100,000 pairs^26^, a recent estimate of the population on the Desertas Islands, Madeira, suggests that the population of this species in Madeira might be almost as large^28^. Elsewhere, populations are smaller^22^.

Using a gene set of 13 anonymous nuclear loci and one mitochondrial locus and coalescence theory-based methods, we studied for the first time the phylogeography and historical demography of Bulwer’s petrel across its main breeding areas in the Atlantic and Pacific Oceans. We assess the level of genetic differentiation and gene flow and provide temporal estimates for population divergence, testing for a possible effect of biogeographical barriers in the evolutionary history of the species. The Atlantic and Pacific Oceans have very different geographical configurations and oceanographic characteristics which determine the complexity of the different biogeographic domains of the two basins^29^. In particular, if we consider their tropical regions, the Atlantic Ocean is a much smaller and enclosed basin with a single predominant bio-oceanographic domain. Conversely, there are at least three recognized biogeographical zones in the much larger Pacific basin characterized by very different biological communities^29^ which might promote isolation and differentiation of seabird populations.

## Results

### Genetic diversity and population structure

A total of 9096 bp were surveyed per individual, including 979 bp of the mitochondrial locus and between 511 and 684 bp of each of the thirteen nuclear loci sampled. (Table 1). Not all individuals sequenced well for all loci, most frequently we sampled ca. 15 individuals for the three populations in the Pacific, ca. 20 individuals for the Azores and Selvagens, 4 individuals for the Raso islet (Cabo Verde) and seven individuals from Desertas (Supplementary information Table 1).

**Table 1:** Polymorphic statistics for all the loci sampled in this study. L–length of the locus in bp; N–number of samples; S–number of segregating sites; N_H_–number of haplotypes; *h*–haplotype diversity; *π*–nucleotide diversity.

Locus	L	*N*	*S*	*N*_*H*_	*h*	*π *(± sd)	Indels
Nuclear anonymous loci*							
Bubu2	663	88	16	17	0.731 ± 0.033	0.0015 ± 0.0001	0
Bubu3	666	99	7	9	0.277 ± 0.042	0.0006 ± 0.0001	0
Bubu4	511	96	3	4	0.614 ± 0.017	0.0015 ± 0.0001	1
Bubu5	548	93	8	9	0.728 ± 0.026	0.0023 ± 0.0001	1
Bubu6	684	100	6	7	0.478 ± 0.032	0.0008 ± 0.0001	0
Bubu7	599	100	8	7	0.643 ± 0.023	0.0019 ± 0.0002	0
Bubu8	619	98	11	13	0.782 ± 0.017	0.0044 ± 0.0001	3
Bubu9	589	100	14	14	0.696 ± 0.029	0.0025 ± 0.0002	1
Bubu10	743	90	11	10	0.747 ± 0.016	0.0022 ± 0.0001	0
Bubu11	594	93	4	5	0.255 ± 0.041	0.0005 ± 0.0001	0
Bubu12	682	83	11	15	0.800 ± 0.018	0.0031 ± 0.0001	0
Bubu14	597	106	9	10	0.381 ± 0.041	0.0014 ± 0.0002	0
Bubu15	622	96	10	11	0.465 ± 0.042	0.0012 ± 0.0001	1
	Total = 8117		Total = 118		Mean = 0.584 ± 0.053	Mean = 0.0018 ± 0.0001	
Mitochondrial locus							
NADH2	979	102	40	26	0.842 ± 0.029	0.0059 ± 0.0007	0

*Anonymous loci described in Silva et al.^30^.

For the total of 8117 bp sequenced for the anonymous nuclear loci, there were 118 segregating sites corresponding to an average of 1 SNP per 69 bp. The number of alleles and segregating sites varied among the nuclear loci, and levels of nucleotide diversity varied by almost an order of magnitude (Table 1). Four of the 13 nuclear loci had length polymorphisms, one of which (an 1 bp indel in the nuclear locus Bubu4) was fixed in the Japanese population. The other three were private to either the Atlantic or Pacific Oceans but were not fixed. In the mitochondrial locus, 40 segregating sites in the 102 sampled individuals defined 26 different haplotypes. Overall, NADH2 was the most polymorphic locus, showing the highest number of segregating sites, haplotypes and nucleotide diversity (*π* = 0.059) (Table 1). For most loci and populations, there were no significant deviations from neutral expectations as suggested by non-significant Tajima’s *D* and Fu’s *F*_*s*_ estimates (Supplementary Table 1). Nuclear and mitochondrial sequence data have been submitted to the GenBank database (accession numbers ON918736-0N920126).

**Figure 1:**
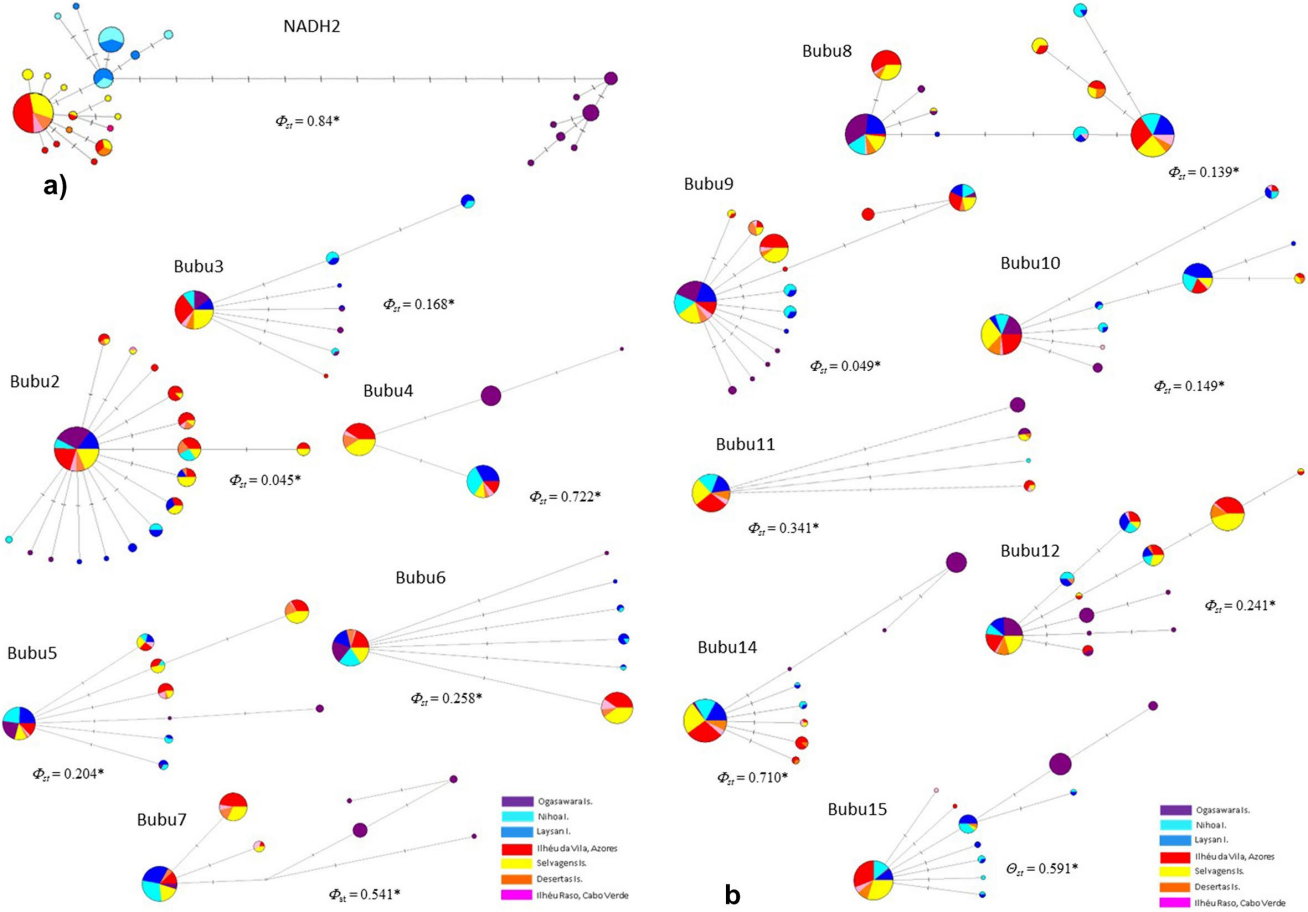
Genetic networks. Medium-joining haplotype networks of the 14 markers surveyed, including the mitochondrial locus NADH2 (**a**) and the thirteen anonymous nuclear loci, as produced by NETWORK v.4.6.0.0 (https://www.fluxus-engineering.com/sharenet.htm). The size of each circle is proportional to the frequency of that haplotype in the sample. The length of each branch is proportional to the number of substitutional steps separating haplotypes.

A very strong pattern of population genetic structure was revealed by the mitochondrial network, with the haplotypes distributed in three clearly segregated groups and no allele sharing between them (Fig. 1a). The group formed by the haplotypes sampled in the Western Pacific was deeply divergent (thirteen substitutions apart) from the clades sampled in the Central Pacific and Atlantic Ocean, corresponding to 1.6% and 1.4% net sequence divergence, respectively. The latter two groups also did not share alleles although haplotypes were much less divergent (0.24% sequence divergence). Within the Atlantic basin, haplotypes were shared among most populations, although the distance between the two colonies farthest apart (Vila islet, Azores and Raso Islet, Cabo Verde) is 2253 km. The global estimate of *Φ*_*st*_ for this locus was high and statistically significant in agreement with the overall geographically structured distribution of the alleles (Fig. 1a).

In contrast, allele networks for the different nuclear loci showed a variable pattern of allele sharing among populations. In several networks, the most common allele was widely distributed and shared among the three main groups but there were also private alleles to the different populations, suggesting some phylogeographic structure. For two loci (Bubu 4 and 11) the alleles sampled in Japan were not shared by any other population (Fig. 1b). Global estimates of *Φ*_*st*_ for the anonymous locus varied between 0.045 (Bubu 2) and 0.722 (Bubu 4) and were significant for all loci. Population pairwise tests (based on *Φ*_*st*_) for the mitochondrial locus suggests a significant genetic differentiation between ocean basins and also between the western and central population of Bulwer’s petrels in the Pacific Ocean (Table 2). The pairwise *Φ*_*st*_ estimates for most nuclear loci reveal a significant differentiation between the Western Pacific and remaining groups (Supplementary Table 2), and only a few suggest significant genetic differentiation between the Central Pacific and the Atlantic populations (Supplementary Table 2). There was no genetic differentiation among the different populations from the Atlantic Ocean suggested by either marker type.

**Table 2:** Pairwise genetic differentiation of Bulwer’s Petrels, estimated by *Φ*_*st*_, between oceans and among island groups within oceans based on the mitochondrial locus NADH2.

	Atlantic ocean				Pacific ocean		
	Azores	Desertas	Selvagens	Cape Verde	Laysan	Nihoa	Japan
Desertas	0.097						
Selvagens	0.001	0.064					
Cape Verde	0.014	0.155	− 0.523				
Laysan I	0.714**	0.694**	0.653**	0.678**			
Nihoa I	0.712**	0.678**	0.655**	0.656**	− 0.001		
Japan	0.941**	0.929**	0.926**	0.927**	0.923**	0.917**	

*P < 0.05, **P < 0.001.

**Figure 2:**
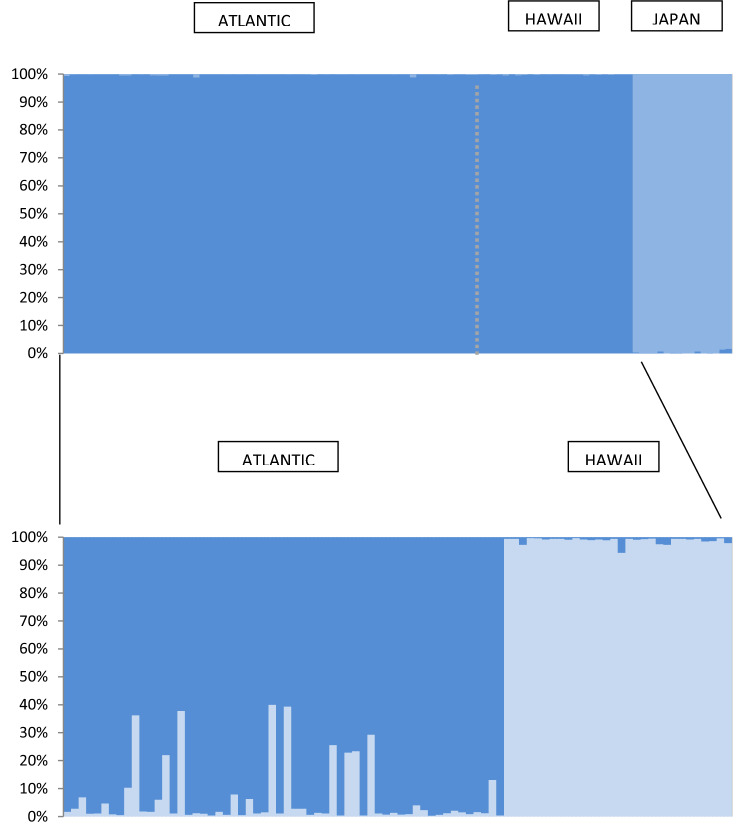
Structure results. Bayesian assignments of co-ancestry for all sampled *Bulweria bulwerii* individuals based on nuclear anonymous genotypes. For all samples, the inferred best *K* by the Evanno et al.^31^ method was two clusters, in both cases.

The STRUCTURE analysis based on the nuclear loci suggested that *K* = 2 best fitted the data, as suggested by *ΔK* (Fig. 2, see also Supplementary Fig. 1) (Evanno et al.^31^) indicating two clearly different genetic clusters, one including all individuals sampled in the Japanese islands, and a second including those of the remaining populations sampled (median posterior probability = 0.99). When STRUCTURE was re-run on the Central Pacific and Atlantic populations alone, it further suggested two genetic clusters corresponding to these two groups, although the Atlantic cluster includes a few individuals with an admixed ancestry (Fig. 2), dispersed throughout the different populations.

## Phylogenetic relationships among major clusters and the historical demography

**Figure 3:**
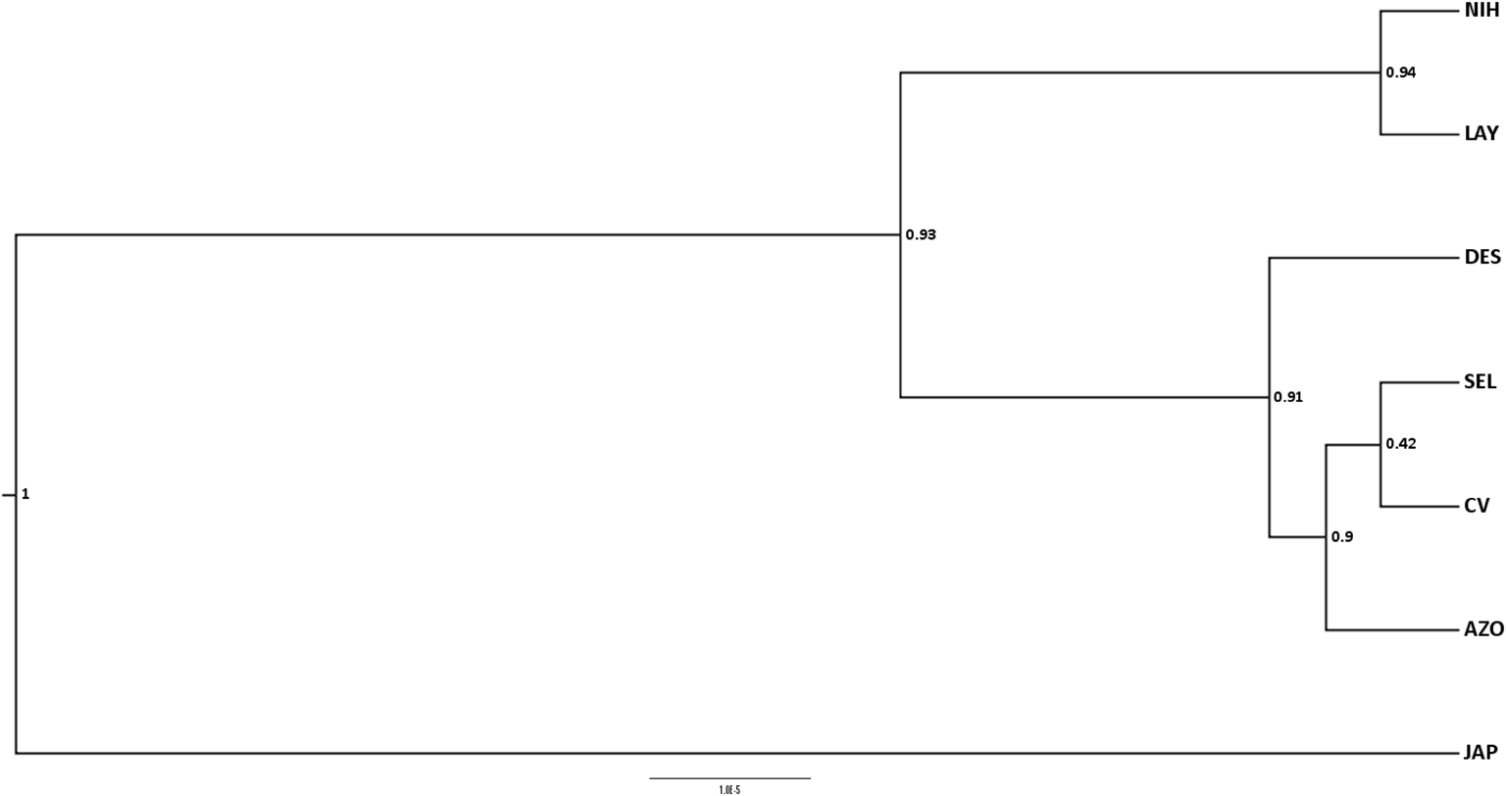
Species tree phylogeny. Phylogeny of the different populations sampled reconstructed with a combined dataset of a mitochondrial locus (NADH2) and thirteen nuclear anonymous markers with BEAST v1.7 (http://beast.community/index.html) and visualized with FigTree v.1.3.1 (http://tree.bio.ed.ac.uk/software/figtree/). Nodal support is indicated by Bayesian posterior probabilities. Bar is in substitutions/site. Abbreviations as follow: AZO–Ilhéu da Vila, Azores; DES–Deserta Grande, Desertas Is; SEL–Selvagem Grande, Selvagens Is., CV–Raso islet, Cabo Verde; LAY–Laysan Island, Hawaii; NIH–Nihoa Island, Hawaii; JAP–Minamijima Is, Ogasawara Islands.

The *BEAST analyses using the mitochondrial and nuclear datasets combined provided strong support for the same topology across replicate runs and suggested a basal group of Bulwer’s petrels from the Western Pacific, and a well-supported sister relationship between the Central Pacific populations and those sampled in the Atlantic Ocean (Fig. 3). The relationships of the different populations within the Atlantic clade are not well resolved.

Implementation of the IMa2 model to three main genetic groups of Bulwer’s petrels, suggested by the BEAST analysis with the combined dataset, and the STRUCTURE analysis, obtained convergence, low autocorrelation values and posterior density curves that showed clear peaks for all demographic parameters estimated (Supplementary Fig. 2). Based on the estimated peaks for *θ*, we inferred a smaller effective population size for the ancestral population of all groups (*N*_*e*_ ~ 9000) and the largest *N*_*e*_ for the ancestral population of the Central Pacific and Atlantic groups, and for the current Central Pacific group, both estimated at approximately 30,000 individuals (Table 3, Supplementary Fig. 2). Regarding the timing of divergences, *t*_*0*_ peaked at 0.110 and *t*_*1*_ peaked at 0.585 (95% HPD not estimated with confidence), suggesting that the Central Pacific and Atlantic groups diverged ~ 160,000 ya, while their ancestor and the Western Pacific group diverged much earlier (~ 850,000 ya) (Table 3, Supplementary Fig. 2). Most of the migration parameter estimates peaked near zero and include zero in their HPD suggesting that these major genetic groups diverged in isolation and have since exchanged very few migrants (Table 3). The only exceptions are the estimate of the migration parameter from the Central Pacific group into the Atlantic, which peaked at 1.744 (95% HPD 0–5.934), suggesting an exchange of ~ 0.6 migrants per generation. Also, *m*_Japan > Hawaii_ peaked at 0.53, suggesting a rare westerly migration of ~ 0.2 migrants per generation (Table 3, Supplementary Fig. 2).

**Table 3:** Estimates of demographic parameters as estimated under an Isolation with Migration model. For the calculation of demographic units, we considered a generation time of 12 years, and a substitution rate per locus (*NADH*_*2*_) of 2.8 × 10^–8^. For the migration parameters, the first population shown is the recipient, and the second population the donor. The 95% highest posterior densities (HPD95) are shown for each parameter.

	Scaled by μ			Demographic units		
Parameter	High point	HPD95High	HPD95Low	High point	HPD95High	HPD95Low
*θ*_Atlantic_	0.553	0.848	0.338	16,839	25,829	10,286
*θ*_C Pacific_	0.950	1.496	0.614	28,938	45,579	18,698
*θ*_W Pacific_	0.623	0.968	0.350	18,972	29,487	10,652
*θ*_A0 (ancestral Atlantic-C Pacific)_	0.963	13.74	0*	29,350	418,849	0*
*θ*_A1 (ancestral of all groups)_	0.305	5.735	0*	9296	174,787	0*
*t*_1 (between W Pacific and other two)_	0.585	9.995	0.205	855,802	14,621,775	299,896
*t*_0 (between Central Pacific and Atlantic)_	0.110	2.998?	0.032?	160,189	4,386,533?	46,082?
*m*_Atlantic > Hawaii_	1.744	5.934*	0*	0.612	1.405*	0.035*
*m*_Hawaii > Atlantic_	0.002	0.911	0*	0.032	0.161	0*
*m*_Atlantic > Japan_	0.105	0.585	0*	0.001	0.437	0*
*m*_Japan > Atlantic_	0.002	0.965	0*	< 0.001	0.171	0*
*m*_Hawaii > Japan_	0.001	0.349	0*	< 0.001	0.298	0*
*m*_Japan > Hawaii_	0.530	1.706	0.006*	0.191	0.497	0.007*

? HPD interval may be incorrect due to multiple peaks.

* Posterior density failed to converge near the limit of the prior.

## Discussion

Our results uncovered a contrasting phylogeographic pattern between the two main ocean basins where the species occurs, including an older evolutionary split between two distinct, isolated lineages evolving in the western and central Pacific Ocean, and a third, panmitic group in the Atlantic Ocean. The remarkable genetic differentiation within the Pacific Ocean but also between ocean basins reveal that both physical and paleo-oceanographic barriers have played an important role in the evolutionary history of this highly vagile, pan-tropical species.

In the present study, the pattern of population differentiation inferred from the complete dataset suggest that the Bulwer’s petrel populations from the Atlantic and Pacific Ocean basins are genetically differentiated, as well as those breeding in the Western and Central Pacific groups. The pattern is more clear with the mitochondrial locus whereas the nuclear data suggest a pattern of intermediate polyphyly between the Central Pacific and Atlantic groups. This is likely due to the nuclear lineages incomplete sorting, in line with the general expectation that nuclear loci will rarely show strong support for monophyly in recently diverged populations^11,18,32,33^.

Despite the absence of obvious physical barriers between Japan and Hawaii, we found two deep evolutionary lineages of Bulwer’s Petrels breeding in these archipelagos, which have been evolving independently for many generations, historically having exchanged very few to no migrants. This pattern of isolation despite the lack of apparent geographical barriers is counterintuitive if we consider that these pelagic seabirds are extremely mobile and routinely undertake journeys of hundreds or thousands of km during incubation shifts or while migrating to their wintering grounds^23–25^. A possible explanation is that a high level of philopatry associated with adaptation to local environments during the breeding season maintains the isolation of the two main genetic clusters found in the Pacific Ocean. The Ogasawara Islands lay in the western subtropical zone of the Pacific and are mostly under the influence of the Kuroshio current, one of its most important currents, which transports warm water northwards along the eastern coast of Japan. Further north, the cold nutrient-rich Oyashio current collides with the Kuroshio current creating a highly productive ecosystem^34^. Archipelagos laying within important current systems, such as the Galapagos or the Cabo Verde, hold higher levels of endemisms and diverged taxa, as species tend to ecologically adapt to local environments^7^. The Ogasawara Islands hold large numbers of pelagic seabirds, and although some also occur in the Northwestern Hawaiian Islands^35^, there are records of seabird population differentiation^36^ and endemisms^37^ suggesting the presence of unique local environmental conditions promoting species diversification.

Other factors may promote population genetic structure in seabirds, including differences in non-breeding areas^6,19^. Although data on species at-sea distribution are scarce for the Pacific Ocean, Bulwer’s petrels from Hawaii are thought to disperse eastward to the central and eastern Pacific during the non-breeding period^26^. Conversely, Bulwer’s petrels from Japan and other western colonies are thought to disperse SW as there have been sightings of this species south of the Korean Peninsula^38^, and into the Indian Ocean^22^. Likely due to its much larger size, the higher oceanographic complexity of the Pacific Ocean^29^ offers multiple high-quality wintering areas for a pelagic species like Bulwer’s petrel. This might enhance the connectivity of the Bulwer’s populations in the Pacific Ocean, creating a much more structured phylogeographic pattern, as has been shown in other species with genetically differentiated populations occupying distinct non-breeding ranges^19^. Conversely, Bulwer’s petrels breeding in the Macaronesian islands of Azores, Madeira, Canaries and Cabo Verde mostly overlap in their wintering areas, in the mid-equatorial Atlantic Ocean^23^. In the Atlantic Ocean, gene flow among the different archipelagos might be enhanced by shared wintering grounds that will favor intermixing of the populations. Consistent with this^27^ report the finding of two breeders in the small population (*ca*. 50 breeding pairs) from Vila islet (Azores) that had been previously captured and ringed on the Desertas Islands, which currently hold the largest population of Bulwer´s petrels in the Atlantic^28^.

Remarkably, and contrary to the pattern found for most other seabird species breeding in Cabo Verde, which holds several endemic Procellariiformes^39^, the Bulwer’s petrels from this archipelago (at least those from Raso Islet) do not appear to be genetically distinct from their more northern conspecifics. Although our sample size for this population is very small, there is clearly no genetic differentiation for the loci sampled, including the faster evolving NADH2. A larger panel of nuclear SNPs should be used to study in detail current levels of population connectivity between the Atlantic archipelagos. It would be interesting in the future to include individuals from Cima islet in Cabo Verde, where the breeding phenology of the species is very different^23^.

There is a clear phylogeographic break between the populations of *B. bulwerii* on either side of the Isthmus of Panama. Petrels from the Hawaiian and Atlantic islands share no mitochondrial haplotypes, and even show significant pairwise differentiation at a few nuclear loci, in a pattern common to many other co-distributed seabirds^9–11,39^. Such replicated phylogeographic patterns across co-distributed taxa suggests similar evolutionary processes in response to common barriers, clearly indicating that the Isthmus of Panama plays a fundamental role in the phylogeography of species with historical distributions in the Atlantic and Pacific. However, in many cases, divergence times suggest that the Pacific and Atlantic populations diverged well after the emergence of the Isthmus of Panama, ~ 3 MYA. Even though continental land bridges represent obvious barriers to dispersal for taxa that do not usually fly over land, the historical demography of many species suggests that this barrier is somewhat permeable, and most species are able of rare dispersal over land^11^.

Interestingly, for many pelagic species including the Bulwer’s petrel, estimates of divergence times suggest that many populations separated during the last 500,000 years^11,32,33,39,40^. The mechanisms by which the glaciation cycles influenced the phylogeographic structure of highly vagile, tropical and sub-tropical seabirds are not well understood. However, the sequential glacial and interglacial periods of the Pleistocene had severe consequences in the global atmospheric conditions and paleo-oceanographic environments^41^, which likely had profound effects on seabird distribution, dispersal patterns, establishment of new populations and extinction of others^42^.

## Conclusion

We describe patterns of genetic differentiation across the main range of Bulwer’s petrel, identify the main phylogeographic breaks and reconstruct its demographic history. Although the Isthmus of Panama has functioned as a barrier to gene flow, we found that oceanographic barriers may also represent critical drivers of diversification in highly pelagic seabird species, despite their ability to travel long distances. In a rapidly changing world, understanding the mechanisms of divergence and speciation at mid-latitudes can provide insights regarding future biological consequences of global change.

The taxonomy of petrels and shearwaters has often been obscured by the lack of obvious morphological variation even between genetically distinct groups^39^. Further genomic, morphological and behavioural data of Bulwer’s petrels from the Ogasawara Islands should be collected as our data argue for a re-examination of the taxonomic status of the species in the Pacific^43^.

## Materials and methods

### Sample and data collection

**Figure 4:**
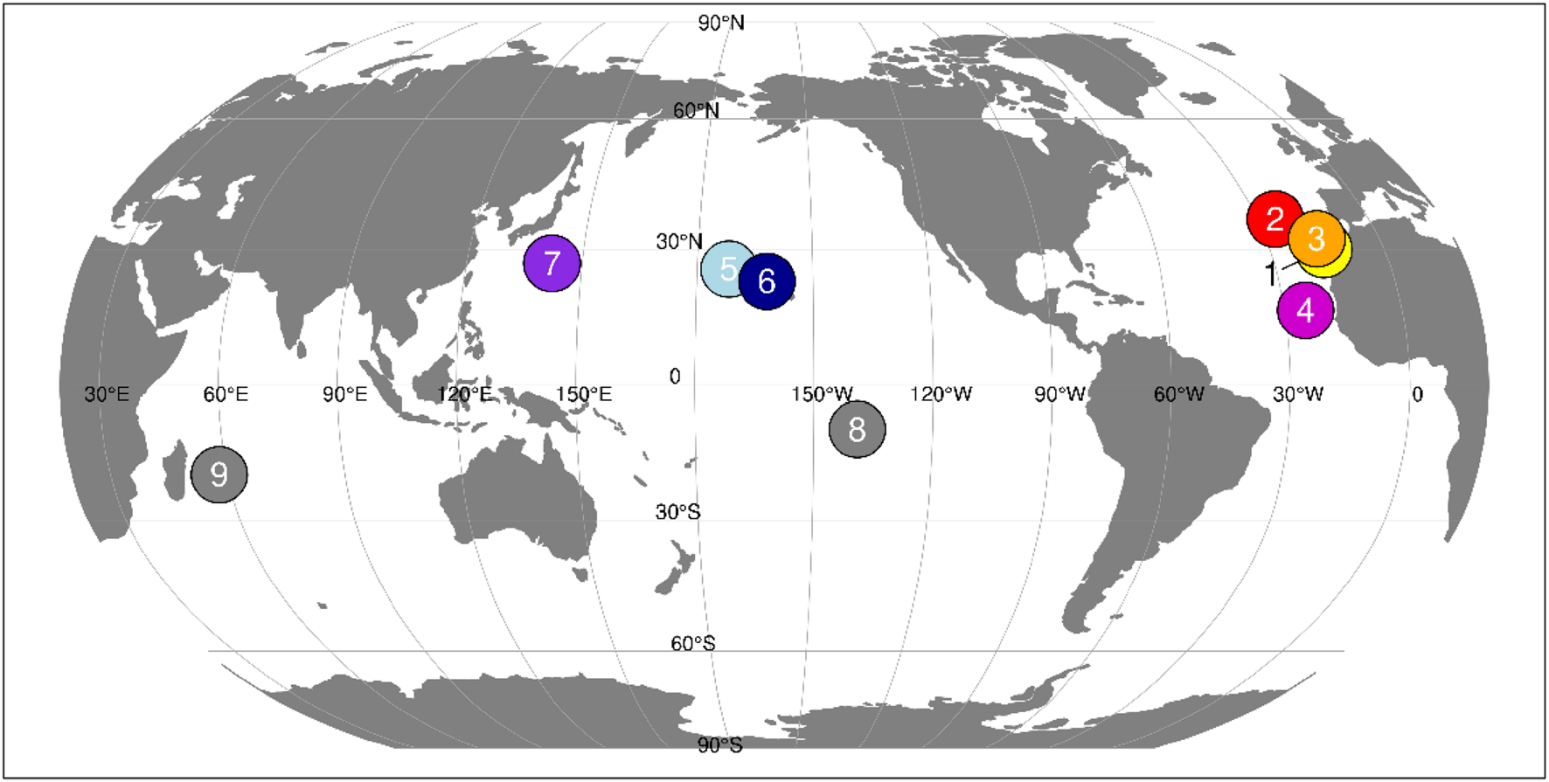
Main breeding areas of Bulwer’s Petrels. In colour are the populations sampled, in light grey are populations not sampled in this study. Sample origin: 1–Selvagem Grande, Selvagens Is., 2—Ilhéu da Vila, Azores; 3–Deserta Grande, Desertas Is; 4–Ilhéu Raso, Cabo Verde; 5–Laysan Is, Hawaii; 6–Nihoa Is, Hawaii; 7—Minamijima Is, Ogasawara Islands; Unsampled: 8–Marquesas Islands; 9–Round Island, Mauritius.

We sampled 105 Bulwer’s petrels from the main breeding localities of the species (Fig. 4). Specifically, we As we were interested in a global phylogeographic perspective, our sampling represents a compromise between the number of populations and loci sampled. Genomic DNA was extracted from blood samples kept in Queen’s lysis buffer^44^ solution following standard phenol–chloroform protocols. We screened for polymorphisms in thirteen anonymous nuclear loci isolated from a genomic library made originally from *B. bulwerii*, as described in^30^. We also surveyed a fragment of the NADH2 locus using primers L5215^45^ and H1064^46^, as the more frequently used Control Region showed a pattern of variation suggesting significant homoplasy (data not shown). Amplification of this fragment was carried out in 25 µl volume reactions containing the same final reagent concentrations as were used with the nuclear loci^30^, as well as the same thermocycler profile but with an annealing temperature of 58 °C. All loci were sequenced in both directions.

Sequences were assembled into contigs, aligned and edited in SEQUENCHER v.4.2 (Gene Codes Corporation), which was also used to check the validity of the base calls. Seven loci displayed heterozygous length polymorphisms, and all gaps from the data sets were trimmed. The phase of nuclear alleles with multiple heterozygous SNPs were resolved using the Bayesian algorithm implemented in PHASE v.2.1.1^47^. Input files were prepared for PHASE using the software SeqPHASE^48^.

### Polymorphism and population structure

Polymorphism statistics, such as the number of segregating sites, haplotypic and nucleotide diversities were determined using DNAsp v5.10^49^. We used ARLEQUIN v.3.5.2.2^50^ to detect departures from neutrality for each population, by estimating Tajima’s *D*^51^ and Fu’s *F*_*S*_^52^ for each locus. The net pairwise sequence divergence between pairs of populations was calculated using MEGA 5^53^ with the model of sequence evolution selected by the AIC in jModelTest^54^. Population structure among the three taxa was investigated by estimating global and pairwise *Ф*_*st*_ for all loci, using the Kimura’s-two parameter model of substitution, as implemented in ARLEQUIN. We tested the significance of results with 10,000 random permutations, considering a significance level of 0.05 after correction for false discovery rate. Median-joining haplotype networks, based on the largest blocks of non-recombining fragments, were estimated for all loci using NETWORK v.4.6.0.0^55^ (https://www.fluxus-engineering.com/sharenet.htm).

To detect population structure without the need to define a priori the populations, we used Bayesian clustering analyses, as implemented in STRUCTURE 2.3^56^ (https://web.stanford.edu/group/pritchardlab/structure.html). We ran two sets of analyses depending on how the data was formatted: the first set used allelic data for each of the nuclear locus, and the second used the dataset converted to SNPs. There were no differences in results between the two sets and we present results only for the former dataset. In the final analysis, In STRUCTURE, colony of origin was not used as prior information, and analyses assumed an admixture model and correlated allele frequencies among populations. We performed 10 replicate runs for each *K* value, which varied between 1 (single population) and 6. Each replicate was run for 1 million iterations, with a burn-in of 100,000 iterations. The value of *K* that best fitted the data was estimated using the method of^31^ implemented in STRUCTURE Harvester^57^. Since this method tends to recover the highest hierarchical level of population structure^31^, we re-ran STRUCTURE to test for further structuring, using the same run parameters as above.

### Phylogenetic relationships among groups

We used the *BEAST algorithm based on the multi-species coalescent^58^ as implemented in the software BEAST 1.7^59^ to estimate a species tree for the sampled taxa, using the mitochondrial and nuclear loci. The recovered topologies of the tree were the same across replicates. As most species trees methods, *BEAST requires a priori assignment of alleles to taxa. We used the well-supported and geographically structured mtDNA network for prior assignment. The input was prepared with BEAUti v1.7 (http://evomics.org/resources/software/molecular-evolution-software/beauti/). We ran *BEAST multiple times to test different priors and settings, and to assess convergence and mixing. The results shown are based on final runs which ran for 10^9^ generations (convergence was compromised by low signal of some genes, and required very long runs), sampling every 10,000 generations, with a burn-in of 10^6^ generations. We chose the most appropriate model of sequence evolution for each locus (see above), used a strict molecular clock, which could not be rejected for this dataset, and a Yule process for the species tree prior, and other priors were changed according to operator weight suggestions from previous runs. We monitored convergence of the sampling process using TRACER 1.5^60^, ensuring that the ESS values were above 200 and parameter posterior distributions were unimodal. Trees were summarized in TreeAnnotator v.1.7^61^ and visualized in FigTree 1.3.1 (http://tree.bio.ed.ac.uk/software/figtree/). We assessed nodal support using posterior probability estimates. We used a substitution rate for the NADH2 locus of 0.028 × 10^–6^^46^, and the nuclear rate was allowed to be estimated relative to the mtDNA rate.

### Coalescence estimates of historical demography

We inferred the demographic history of Bulwer’s Petrel with IMa2^62^ using the full data set of twelve anonymous nuclear and NADH2 loci. IMa2 implements a coalescent-based method that uses Markov Chain Monte Carlo sampling of gene genealogies to estimate population parameters scaled by the mutation rate per locus per generation (*μ*), such as contemporary and ancestral effective population sizes (*θ*, where *θ* = *4N*_*e*_*μ*, and *N*_*e*_ is the effective population size) migration rates (*m* = *M/μ*, where *M* is the effective migration rate per generation) and divergence times (*t* = *Tμ*, where *T* is the divergence time in years before present) among populations. Implementation of IMa2 with more than two populations requires a known sequence of splitting events, and accordingly we used our *BEAST species tree. It also assumes no recombination within loci, and we used IMgc^63^ to find the largest block of sequences and individuals with no evidence of recombination and used these data in our IMa2 analysis. We ran IMa2 several times, varying the prior bounds, heating schemes and number of chains, to optimize the priors, improve mixing of the chains and reach convergence of the different analyses. The final runs included 80 independent MCMC chains with a geometric heating scheme (*h*1 = 0.975, *h*2 = 0.75). All runs included 15 million generations with 500,000 generations discarded as burn-in. Trend plots, ESS values and comparisons between coupled runs were used to assess mixing and convergence, as suggested in the documentation for IMa2. To translate parameter estimates (scaled by *µ*) to demographic units, we used a substitution rate for NADH2 of 0.028 × 10^–6^^46^, and a mean generation time of 12 years^64^.

Ethical statement. The volume of the blood sample collected from each petrel was 50–75 μl (approximately 0.05% vol/weight). Sampling were performed in accordance with the guidelines and regulations of each of the relevant institutions, including the Instituto das Florestas e Conservação da Biodiversidade, Portugal (permit No. 2/2012S), the Direção Regional dos Açores (permits nº4/CN/2002 and nº7/CN/2003), the US Fish and Wildlife Services and the Papahānaumokuākea Marine National Monument (permit No. PMNM-2008-029) and the Ministry of the Environment, Japan (permit No. 080428001). Sampling further followed the requirements of the Directive 2010/63/EU of the European Parliament and of the council for the protection of animals used for scientific purposes.

## Supplementary Information

Supplementary Information.

## Data Availability

DNA sequences: GenBank accession #s: ON918736–ON920126. While being curated by GenBank, the sequence data can be found temporarily at https://drive.google.com/drive/folders/1Gwhfiro2aq7aSwLTJmi2cn7Vgh2bz0UM?usp=sharing. For additional information contact corresponding author (MCS).
