# Supplementary material for: Contrasting patterns of population structure of Bulwer’s petrel (Bulweria bulwerii) between oceans revealed by statistical phylogeography
Source: Sci Rep. 2023 Feb 2;13:1939. doi: 10.1038/s41598-023-28452-z (PMC9895040; doi:10.1038/s41598-023-28452-z)
Supplement: Supplementary file 1 — Supplementary Information. [file 41598_2023_28452_MOESM1_ESM.docx]

**Supplementary information**

**Contrasting patterns of population structure of Bulwer’s petrel (*Bulweria bulwerii*) between oceans revealed by statistical phylogeography**

Mónica C. Silva^1^, Paulo Catry^2^, Joël Bried^3,*^, Kazuto Kawakami^4^, Elizabeth Flint^5^, José P. Granadeiro^6^

Appendix Table 1. N is the number of samples, S the number of segregating sites, N_H_ the number of haplotypes, *h* is the haplotype diversity and *π* the nucleotide diversity. We used ARLEQUIN v.3.5.2 to detect departures from neutrality for each population, by estimating Tajima’s *D* (Tajima 1989) and Fu’s *F_S_* (Fu 1997) for each locus. *Significance levels were *P* < 0.05 for Tajima’s *D* and *P* < 0.02 for Fu’s *F_S_*.

| **Locus** | **Population** | **N** | **S** | **N_H_** | ***h*** | ***π*** | ***D*** | ***Fs*** |
| --- | --- | --- | --- | --- | --- | --- | --- | --- |
| **Bubu2** | **Cabo Verde** | 3 | 2 | 3 | 0.600 ± 0.215 | 0.0010 ± 0.0004 | -1.13 | -0.86 |
|  | **Selvagens** | 18 | 8 | 9 | 0.767 ± 0.060 | 0.0017 ± 0.0002 | -1.17 | -4.16* |
|  | **Desertas** | 7 | 4 | 5 | 0.758 ± 0.084 | 0.0015 ± 0.0003 | -0.7 | -1.66 |
|  | **Azores** | 26 | 8 | 9 | 0.832 ± 0.035 | 0.0019 ± 0.0002 | -0.75 | -2.91 |
|  | **Nihoa** | 8 | 3 | 4 | 0.758 ± 0.060 | 0.0015 ± 0.0002 | 0.36 | -0.22 |
|  | **Laysan** | 13 | 6 | 7 | 0.729 ± 0.082 | 0.0014 ± 0.0002 | -1.19 | -3.24* |
|  | **Japan** | 13 | 2 | 3 | 0.195 ± 0.115 | 0.0003 ± 0.0002 | -1.51* | -1.86* |
|  |  |  |  |  |  |  |  |  |
| **Bubu3** | **Cabo Verde** | 4 | 0 | 1 | 0.000 ± 0.000 | 0.0000 ± 0.0000 | 0 | - |
|  | **Selvagens** | 21 | 0 | 1 | 0.000 ± 0.000 | 0.0000 ± 0.0000 | 0 | - |
|  | **Desertas** | 6 | 0 | 1 | 0.000 ± 0.000 | 0.0000 ± 0.0000 | 0 | - |
|  | **Azores** | 24 | 1 | 2 | 0.042 ± 0.040 | 0.0001 ± 0.0001 | -1.11 | -1.6 |
|  | **Nihoa** | 15 | 3 | 5 | 0.648 ± 0.083 | 0.0013 ± 0.0002 | 0.38 | -0.97 |
|  | **Laysan** | 15 | 4 | 6 | 0.644 ± 0.082 | 0.0015 ± 0.0002 | -0.09 | -1.73 |
|  | **Japan** | 14 | 2 | 3 | 0.255 ± 0.116 | 0.0004 ± 0.0002 | -1.18 | -1.31 |
|  |  |  |  |  |  |  |  |  |
| **Bubu4** | **Cabo Verde** | 4 | 1 | 2 | 0.536 ± 0.123 | 0.0011 ± 0.0002 | 1.17 | 0.87 |
|  | **Selvagens** | 20 | 1 | 2 | 0.385 ± 0.070 | 0.0008 ± 0.0001 | 0.91 | 1.29 |
|  | **Desertas** | 7 | 1 | 2 | 0.440 ± 0.112 | 0.0009 ± 0.0002 | 0.84 | 0.94 |
|  | **Azores** | 21 | 1 | 2 | 0.418 ± 0.061 | 0.0008 ± 0.0001 | 1.13 | 1.47 |
|  | **Nihoa** | 15 | 0 | 1 | 0.000 ± 0.000 | 0.0000 ± 0.0000 | 0 | - |
|  | **Laysan** | 15 | 0 | 1 | 0.000 ± 0.000 | 0.0000 ± 0.0000 | 0 | - |
|  | **Japan** | 14 | 0 | 1 | 0.000 ± 0.000 | 0.0000 ± 0.0000 | 0 | - |
|  |  |  |  |  |  |  |  |  |
| **Bubu5** | **Cabo Verde** | 4 | 4 | 4 | 0.821 ± 0.101 | 0.0027 ± 0.0007 | -0.22 | -0.47 |
|  | **Selvagens** | 20 | 4 | 5 | 0.753 ± 0.041 | 0.0025 ± 0.0001 | 1.08 | 0.54 |
|  | **Desertas** | 5 | 3 | 3 | 0.511 ± 0.164 | 0.0021 ±0.0007 | 0.25 | 0.72 |
|  | **Azores** | 21 | 4 | 5 | 0.794 ± 0.024 | 0.0027 ± 0.0002 | 1.38 | 0.81 |
|  | **Nihoa** | 15 | 4 | 5 | 0.545 ± 0.101 | 0.0012 ± 0.0003 | -0.97 | -1.86 |
|  | **Laysan** | 15 | 3 | 4 | 0.494 ± 0.100 | 0.0010 ± 0.0002 | -0.63 | -0.98 |
|  | **Japan** | 13 | 2 | 3 | 0.385 ± 0.113 | 0.0012 ± 0.0004 | 0.54 | 0.46 |
|  |  |  |  |  |  |  |  |  |
| **Bubu6** | **Cabo Verde** | 4 | 1 | 2 | 0.429 ± 0.169 | 0.0006 ± 0.0003 | 0.33 | 0.54 |
|  | **Selvagens** | 21 | 1 | 2 | 0.512 ± 0.017 | 0.0008 ± 0.0000 | 1.7 | 1.89 |
|  | **Desertas** | 7 | 1 | 2 | 0.440 ± 0.112 | 0.0006 ± 0.0002 | 0.84 | 0.94 |
|  | **Azores** | 24 | 1 | 2 | 0.503 ± 0.023 | 0.0007 ± 0.0000 | 1.67 | 1.93 |
|  | **Nihoa** | 15 | 3 | 4 | 0.193 ± 0.095 | 0.0003 ± 0.0002 | -1.73* | -3.38* |
|  | **Laysan** | 15 | 4 | 5 | 0.492 ± 0.100 | 0.0008 ± 0.0002 | -1.17 | -2.23 |
|  | **MI** | 3 | 0 | 1 | 0.000 ± 0.000 | 0.0000 ± 0.0000 | 0 | - |
|  | **Japan** | 11 | 1 | 2 | 0.091 ± 0.081 | 0.0001 ± 0.0001 | -1.16 | -0.96* |
|  |  |  |  |  |  |  |  |  |
| **Bubu7** | **Cabo Verde** | 4 | 2 | 2 | 0.571 ± 0.094 | 0.0019 ± 0.0003 | 1.79 | 2.22 |
|  | **Selvagens** | 21 | 2 | 3 | 0.595 ± 0.037 | 0.0012 ± 0.0001 | 0.89 | 0.97 |
|  | **Desertas** | 7 | 1 | 2 | 0.495 ± 0.088 | 0.0008 ± 0.0002 | 1.21 | 1.14 |
|  | **Azores** | 22 | 2 | 3 | 0.469 ± 0.065 | 0.0009 ± 0.0002 | 0.29 | 0.43 |
|  | **Nihoa** | 15 | 0 | 1 | 0.000 ± 0.000 | 0.0000 ± 0.0000 | 0 | - |
|  | **Laysan** | 15 | 0 | 1 | 0.000 ± 0.000 | 0.0000 ± 0.0000 | 0 | - |
|  | **Japan** | 16 | 6 | 5 | 0.714 ± 0.082 | 0.0026 ± 0.0005 | -0.18 | 0.14 |
|  |  |  |  |  |  |  |  |  |
| **Bubu8** | **Cabo Verde** | 4 | 5 | 4 | 0.643 ± 0.184 | 0.0034 ± 0.0012 | 0.34 | 0.23 |
|  | **Selvagens** | 21 | 8 | 6 | 0.803 ± 0.028 | 0.0050 ± 0.0003 | 1.89 | 2.6 |
|  | **Desertas** | 7 | 6 | 5 | 0.824 ± 0.057 | 0.0046 ± 0.0005 | 1.78 | 0.96 |
|  | **Azores** | 23 | 7 | 5 | 0.674 ± 0.040 | 0.0047 ± 0.0003 | 2.19 | 3.57 |
|  | **Nihoa** | 15 | 6 | 5 | 0.768 ± 0.036 | 0.0040 ± 0.0004 | 1.76 | 2.01 |
|  | **Laysan** | 15 | 6 | 5 | 0.639 ± 0.052 | 0.0035 ± 0.0003 | 1.26 | 1.61 |
|  | **Japan** | 13 | 2 | 3 | 0.450 ± 0.112 | 0.0008 ± 0.0002 | -0.26 | -0.2 |
|  |  |  |  |  |  |  |  |  |
| **Bubu9** | **Cabo Verde** | 4 | 2 | 3 | 0.607 ± 0.164 | 0.0012 ± 0.0004 | -0.45 | -0.48 |
|  | **Selvagens** | 21 | 6 | 5 | 0.691 ± 0.045 | 0.0023 ± 0.0004 | -0.03 | 0.61 |
|  | **Desertas** | 7 | 5 | 4 | 0.747 ± 0.078 | 0.0025 ± 0.0007 | -0.17 | 0.48 |
|  | **Azores** | 22 | 7 | 7 | 0.779 ± 0.035 | 0.0037 ± 0.0003 | 0.99 | 0.46 |
|  | **Nihoa** | 15 | 5 | 4 | 0.637 ± 0.078 | 0.0023 ± 0.0005 | 0.17 | 1.18 |
|  | **Laysan** | 15 | 6 | 5 | 0.536 ± 0.097 | 0.0020 ± 0.0005 | -0.62 | -0.18 |
|  | **Japan** | 16 | 4 | 5 | 0.338 ± 0.128 | 0.0006 ± 0.0003 | -1.88* | -3.82* |
|  |  |  |  |  |  |  |  |  |
| **Bubu10** | **Cabo Verde** | 3 | 7 | 4 | 0.800 ± 0.172 | 0.0034 ± 0.0011 | -1.01 | -0.07 |
|  | **Selvagens** | 19 | 4 | 4 | 0.647 ± 0.044 | 0.0016 ± 0.0003 | 0.53 | 1.03 |
|  | **Desertas** | 7 | 4 | 3 | 0.604 ± 0.076 | 0.0013 ± 0.0005 | -0.79 | 0.76 |
|  | **Azores** | 19 | 7 | 5 | 0.684 ± 0.058 | 0.0022 ± 0.0004 | -0.01 | 1.06 |
|  | **Nihoa** | 14 | 7 | 6 | 0.794 ± 0.041 | 0.0026 ± 0.0003 | 0.27 | 0.23 |
|  | **Laysan** | 15 | 8 | 7 | 0.664 ± 0.089 | 0.0025 ± 0.0005 | -0.2 | -0.63 |
|  | **Japan** | 13 | 1 | 2 | 0.312 ± 0.106 | 0.0004 ± 0.0001 | 0.24 | 0.65 |
|  |  |  |  |  |  |  |  |  |
| **Bubu11** | **Cabo Verde** | 4 | 1 | 2 | 0.250 ± 0.180 | 0.0004 ± 0.0003 | -1.05 | -0.18 |
|  | **Selvagens** | 21 | 2 | 3 | 0.180 ± 0.077 | 0.0003 ± 0.0001 | -1.12 | -1.61 |
|  | **Desertas** | 7 | 1 | 2 | 0.143 ± 0.119 | 0.0002 ± 0.0002 | -1.16 | -0.6 |
|  | **Azores** | 25 | 1 | 2 | 0.150 ± 0.065 | 0.0003 ± 0.0001 | -0.44 | -0.08 |
|  | **Nihoa** | 15 | 1 | 2 | 0.067 ± 0.061 | 0.0001 ± 0.0001 | -1.15* | -1.21 |
|  | **Laysan** | 13 | 0 | 1 | 0.000 ± 0.000 | 0.0000 ± 0.0000 | 0 | - |
|  | **Japan** | 8 | 2 | 3 | 0.591 ± 0.108 | 0.0017 ± 0.0003 | 1.56 | 0.72 |
|  |  |  |  |  |  |  |  |  |
| **Bubu12** | **Cabo Verde** | 2 | 5 | 4 | 1.000 ± 0.177 | 0.0039 ± 0.0011 | -0.21 | -1.41 |
|  | **Selvagens** | 21 | 6 | 7 | 0.702 ± 0.054 | 0.0027 ± 0.0003 | 0.81 | -0.23 |
|  | **Desertas** | 7 | 4 | 4 | 0.670 ± 0.082 | 0.0026 ± 0.0003 | 1.28 | 0.85 |
|  | **Azores** | 21 | 7 | 7 | 0.763 ± 0.044 | 0.0031 ± 0.0003 | 0.84 | 0.28 |
|  | **Nihoa** | 8 | 4 | 4 | 0.792 ± 0.045 | 0.0023 ± 0.0004 | 0.98 | 0.81 |
|  | **Laysan** | 10 | 4 | 5 | 0.779 ± 0.052 | 0.0024 ± 0.0003 | 1.32 | 0.16 |
|  | **Japan** | 14 | 5 | 6 | 0.719 ± 0.065 | 0.0015 ± 0.0003 | -0.7 | -1.96 |
|  |  |  |  |  |  |  |  |  |
| **Bubu14** | **Cabo Verde** | 4 | 1 | 2 | 0.250 ± 0.180 | 0.0004 ± 0.0003 | -1.05 | -0.18 |
|  | **Selvagens** | 21 | 1 | 2 | 0.048 ± 0.045 | 0.0001 ± 0.0001 | -1.12* | -1.49 |
|  | **Desertas** | 7 | 2 | 3 | 0.275 ± 0.148 | 0.0005 ± 0.0003 | -1.48 | -1.48 |
|  | **Azores** | 29 | 3 | 4 | 0.328 ± 0.073 | 0.0006 ± 0.0001 | -0.94 | -1.6 |
|  | **Nihoa** | 15 | 2 | 3 | 0.191 ± 0.093 | 0.0003 ± 0.0002 | -1.26* | -1.67 |
|  | **Laysan** | 15 | 2 | 3 | 0.131 ± 0.082 | 0.0002 ± 0.0001 | -1.51* | -2.36* |
|  | **Japan** | 15 | 4 | 4 | 0.398 ± 0.122 | 0.0016 ± 0.0005 | -0.35 | 0 |
|  |  |  |  |  |  |  |  |  |
| **Bubu15** | **Cabo Verde** | 4 | 1 | 2 | 0.250 ± 0.180 | 0.0004 ± 0.0003 | -1.05 | -0.18 |
|  | **Selvagens** | 21 | 1 | 2 | 0.048 ± 0.045 | 0.0001 ± 0.0001 | -1.12* | -1.49 |
|  | **Desertas** | 7 | 1 | 2 | 0.143 ± 0.119 | 0.0002 ± 0.0002 | -1.16 | -0.59 |
|  | **Azores** | 22 | 1 | 2 | 0.045 ± 0.043 | 0.0001 ± 0.0001 | -1.12 | -1.53* |
|  | **Nihoa** | 15 | 5 | 6 | 0.570 ± 0.092 | 0.0011 ± 0.0002 | -1.22 | -2.78* |
|  | **Laysan** | 13 | 5 | 6 | 0.671 ± 0.070 | 0.0014 ± 0.0002 | -1 | -2.35 |
|  | **Japan** | 14 | 3 | 3 | 0.426 ± 0.122 | 0.0010 ± 0.0004 | -0.63 | 0.29 |
|  |  |  |  |  |  |  |  |  |
| **NADH2** | **Cabo Verde** | 4 | 2 | 2 | 0.500 ± 0.265 | 0.0010 ± 0.0005 | -0.71 | 1.1 |
|  | **Selvagens** | 21 | 8 | 8 | 0.719 ± 0.099 | 0.0013 ± 0.0003 | -1.45 | -3.87* |
|  | **Desertas** | 7 | 2 | 3 | 0.667 ± 0.160 | 0.0011 ± 0.0003 | 1.17 | 0.11 |
|  | **Azores** | 24 | 7 | 6 | 0.442 ± 0.124 | 0.0008 ± 0.0003 | -1.90* | -2.94* |
|  | **Nihoa** | 15 | 5 | 4 | 0.619 ± 0.120 | 0.0013 ± 0.0004 | -0.57 | 0.21 |
|  | **Laysan** | 15 | 4 | 4 | 0.705 ± 0.074 | 0.0011 ± 0.0002 | -0.53 | -0.27 |
|  | **Japan** | 16 | 5 | 6 | 0.855 ± 0.085 | 0.0015 ± 0.0003 | -0.65 | -2.51 |

Appendix Table 2. Pairwise genetic differentiation of Bulwer’s Petrels, estimated by *Φ_st_*, between oceans and among island groups within oceans based on the anonymous nuclear loci.

Locus Bubu2

|  | Azores | Desertas | Selvagens | Cape Verde | Laysan | Nihoa |
| --- | --- | --- | --- | --- | --- | --- |
| Desertas | -0.016 |  |  |  |  |  |
| Selvagens | 0.008 | 0.004 |  |  |  |  |
| Cape Verde | -0.011 | 0.014 | 0.008 |  |  |  |
| Laysan I. | 0.061** | 0.092** | 0.036 | 0.014 |  |  |
| Nihoa I. | 0.048* | 0.015 | 0.061* | 0.095* | 0.098* |  |
| Japan | 0.061** | 0.171** | 0.071** | 0.124 | 0.053** | 0.210** |

**P* < 0.05, ***P* < 0.001

Locus Bubu3

|  | Azores | Desertas | Selvagens | Cape Verde | Laysan | Nihoa |
| --- | --- | --- | --- | --- | --- | --- |
| Desertas | -0.041 |  |  |  |  |  |
| Selvagens | -0.003 | 0.000 |  |  |  |  |
| Cape Verde | -0.065 | 0.000 | 0.000 |  |  |  |
| Laysan I. | 0.286** | 0.154* | 0.281** | 0.120 |  |  |
| Nihoa I. | 0.227** | 0.107* | 0.226** | 0.074 | 0.001 |  |
| Japan | 0.037 | -0.169 | 0.045* | -0.044 | 0.201** | 0.153** |

**P* < 0.05, ***P* < 0.001

Locus Bubu4

|  | Azores | Desertas | Selvagens | Cape Verde | Laysan | Nihoa |
| --- | --- | --- | --- | --- | --- | --- |
| Desertas | -0.050 |  |  |  |  |  |
| Selvagens | -0.022 | -0.047 |  |  |  |  |
| Cape Verde | 0.160 | 0.127 | 0.213 |  |  |  |
| Laysan I. | 0.672** | 0.787** | 0.716** | 0.559* |  |  |
| Nihoa I. | 0.672** | 0.787** | 0.716** | 0.559** | 0.000 |  |
| Japan | 0.793** | 0.849** | 0.805** | 0.892** | 0.983** | 0.983** |

**P* < 0.05, ***P* < 0.001

Locus Bubu5

|  | Azores | Desertas | Selvagens | Cape Verde | Laysan | Nihoa |
| --- | --- | --- | --- | --- | --- | --- |
| Desertas | 0.124* |  |  |  |  |  |
| Selvagens | 0.013 | 0.034 |  |  |  |  |
| Cape Verde | 0.251 | 0.339* | 0.158 |  |  |  |
| Laysan I. | 0.201** | 0.586** | 0.308** | 0.163* |  |  |
| Nihoa I. | 0.166** | 0.539** | 0.267** | 0.144* | -0.021 |  |
| Japan | 0.221** | 0.557** | 0.318** | 0.212** | 0.097** | 0.088** |

**P* < 0.05, ***P* < 0.001

Locus Bubu6

|  | Azores | Desertas | Selvagens | Cape Verde | Laysan | Nihoa |
| --- | --- | --- | --- | --- | --- | --- |
| Desertas | 0.001 |  |  |  |  |  |
| Selvagens | -0.015 | 0.043 |  |  |  |  |
| Cape Verde | 0.111 | 0.284 | 0.048 |  |  |  |
| Laysan I. | 0.289** | 0.148** | 0.338** | 0.514** |  |  |
| Nihoa I. | 0.323** | 0.205** | 0.388** | 0.691** | 0.020 |  |
| Japan | 0.346** | 0.276* | 0.417** | 0.792** | 0.072* | -0.001 |

**P* < 0.05, ***P* < 0.001

Locus Bubu7

|  | Azores | Desertas | Selvagens | Cape Verde | Laysan | Nihoa |
| --- | --- | --- | --- | --- | --- | --- |
| Desertas | -0.042 |  |  |  |  |  |
| Selvagens | 0.048 | 0.010 |  |  |  |  |
| Cape Verde | 0.239* | 0.217* | 0.126 |  |  |  |
| Laysan I. | 0.590** | 0.726** | 0.358** | 0.684** |  |  |
| Nihoa I. | 0.590** | 0.726** | 0.358** | 0.684** | 0.000 |  |
| Japan | 0.677** | 0.615** | 0.622** | 0.578** | 0.662** | 0.662** |

**P* < 0.05, ***P* < 0.001

Locus Bubu8

|  | Azores | Desertas | Selvagens | Cape Verde | Laysan | Nihoa |
| --- | --- | --- | --- | --- | --- | --- |
| Desertas | -0.024 |  |  |  |  |  |
| Selvagens | -0.006 | -0.041 |  |  |  |  |
| Cape Verde | -0.006 | 0.001 | 0.008 |  |  |  |
| Laysan I. | 0.046* | -0.008 | 0.020 | -0.017 |  |  |
| Nihoa I. | 0.069* | 0.040 | 0.057* | -0.037 | -0.002 |  |
| Japan | 0.433** | 0.467** | 0.361** | 0.757** | 0.474** | 0.530** |

**P* < 0.05, ***P* < 0.001

Locus Bubu9

|  | Azores | Desertas | Selvagens | Cape Verde | Laysan | Nihoa |
| --- | --- | --- | --- | --- | --- | --- |
| Desertas | 0.060* |  |  |  |  |  |
| Selvagens | 0.043 | 0.014 |  |  |  |  |
| Cape Verde | 0.096 | -0.017 | -0.017 |  |  |  |
| Laysan I. | 0.095* | 0.035 | 0.054 | 0.073 |  |  |
| Nihoa I. | 0.103** | 0.046 | 0.068* | 0.077 | -0.026 |  |
| Japan | 0.173** | 0.081* | 0.084** | 0.060 | 0.024 | 0.044 |

**P* < 0.05, ***P* < 0.001

Locus Bubu10

|  | Azores | Desertas | Selvagens | Cape Verde | Laysan | Nihoa |
| --- | --- | --- | --- | --- | --- | --- |
| Desertas | 0.016 |  |  |  |  |  |
| Selvagens | -0.003 | -0.024 |  |  |  |  |
| Cape Verde | -0.034 | 0.034 | 0.042 |  |  |  |
| Laysan I. | 0.173** | 0.321** | 0.295** | 0.155 |  |  |
| Nihoa I. | -0.003 | 0.087 | 0.059* | -0.023 | 0.074* |  |
| Japan | 0.132* | 0.061 | 0.108** | 0.192* | 0.449** | 0.210** |

**P* < 0.05, ***P* < 0.001

Locus Bubu11

|  | Azores | Desertas | Selvagens | Cape Verde | Laysan | Nihoa |
| --- | --- | --- | --- | --- | --- | --- |
| Desertas | 0.031 |  |  |  |  |  |
| Selvagens | 0.027 | -0.046 |  |  |  |  |
| Cape Verde | -0.064 | 0.017 | 0.005 |  |  |  |
| Laysan I. | 0.034 | 0.047 | 0.018 | 0.169 |  |  |
| Nihoa I. | 0.035 | 0.016 | 0.021 | 0.078 | -0.005 |  |
| Japan | 0.625** | 0.471** | 0.573** | 0.423** | 0.617** | 0.607** |

**P* < 0.05, ***P* < 0.001

Locus Bubu12

|  | Azores | Desertas | Selvagens | Cape Verde | Laysan | Nihoa |
| --- | --- | --- | --- | --- | --- | --- |
| Desertas | -0.034 |  |  |  |  |  |
| Selvagens | 0.010 | 0.007 |  |  |  |  |
| Cape Verde | -0.064 | -0.066 | 0.092 |  |  |  |
| Laysan I. | 0.208** | 0.228** | 0.369** | -0.040 |  |  |
| Nihoa I. | 0.216* | 0.237* | 0.380* | -0.028 | -0.056 |  |
| Japan | 0.361** | 0.396** | 0.506** | 0.313** | 0.278** | 0.380** |

**P* < 0.05, ***P* < 0.001

Locus Bubu14

|  | Azores | Desertas | Selvagens | Cape Verde | Laysan | Nihoa |
| --- | --- | --- | --- | --- | --- | --- |
| Desertas | -0.027 |  |  |  |  |  |
| Selvagens | 0.067* | 0.052 |  |  |  |  |
| Cape Verde | 0.024 | -0.003 | 0.055 |  |  |  |
| Laysan I. | 0.055* | 0.016 | 0.005 | 0.034 |  |  |
| Nihoa I. | 0.060* | 0.018 | 0.027 | 0.023 | -0.027 |  |
| Japan | 0.831** | 0.795** | 0.875** | 0.782** | 0.842** | 0.833** |

**P* < 0.05, ***P* < 0.001

Locus Bubu15

|  | Azores | Desertas | Selvagens | Cape Verde | Laysan | Nihoa |
| --- | --- | --- | --- | --- | --- | --- |
| Desertas | 0.034 |  |  |  |  |  |
| Selvagens | 0.000 | -0.016 |  |  |  |  |
| Cape Verde | 0.126 | 0.017 | 0.119 |  |  |  |
| Laysan I. | 0.261** | 0.079* | 0.227** | 0.108 |  |  |
| Nihoa I. | 0.149** | 0.012 | 0.118** | 0.046 | -0.009 |  |
| Japan | 0.882** | 0.798** | 0.877** | 0.785** | 0.639** | 0.687** |

**P* < 0.05, ***P* < 0.001


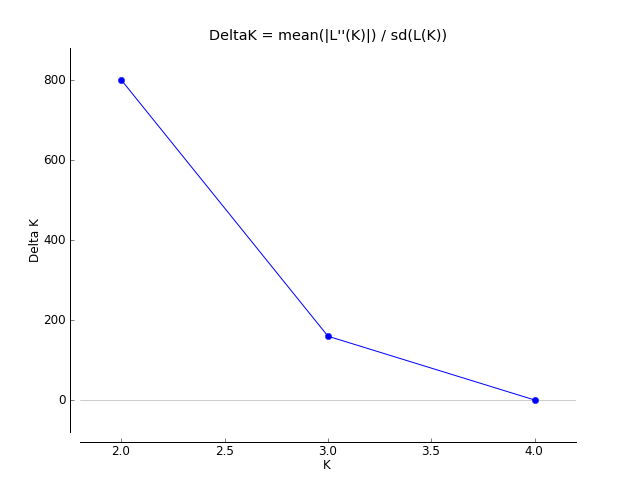


Appendix Figure 1. Structure *delta* K plot for finding best fit K for the input data using the Evanno (2005) method.

a)

Time (*t*)

Posterior probability

b)

Posterior probability

Effective population size (*θ*)

c)

Posterior probability

Effective population size (*θ*)

d)

Posterior probability

Migration (*m*)

e)

Posterior probability

Migration (*m*)

f)

Migration (*m*)

Posterior probability

Appendix Figure 2. Posterior distributions for the different population parameters estimated with IMa2. All the parameter estimates are scaled by the mutation rate (*μ*). a) through f) show results from a final run, which used 13 markers and settings as described in the text.

References

Evanno, G., Regnaut, S., Goudet, J. (2005) Detecting the number of clusters of individuals using the software STRUCTURE: a simulation study. *Molecular Ecology*, **14**, 2611-2620. <https://doi.org/10.1111/j.1365-294X.2005.02553.x>

Excoffier, L., Laval, G., Schneider, S. (2005) Arlequin v. 3.0: An integrated software package for population genetics data analysis. *Evolutionary Bioinformatics Online*, **1**, 47-50. [https://doi.org/10.1177/117693430500100003](https://doi.org/10.1177%2F117693430500100003)

Fu, Y.X. (1997). Statistical tests of neutrality of mutations against population growth, hitchhiking and background selection. *Genetics,* **147**, 915-925. <https://doi.org/10.1093/genetics/147.2.915>

Tajima, F. (1989). Statistical method for testing the neutral mutation hypothesis by DNA polymorphism. *Genetics*, **123**,585-595. <https://doi.org/10.1093/genetics/123.3.585>
